# *In Silico* Screening of Circulating MicroRNAs as Potential Biomarkers for the Diagnosis of Ovarian Cancer

**DOI:** 10.1155/2019/7541857

**Published:** 2019-08-04

**Authors:** Lei Wu, Wenwen Shang, Hong Zhao, Guodong Rong, Yan Zhang, Ting Xu, Jiexin Zhang, Peijun Huang, Fang Wang

**Affiliations:** ^1^Department of Laboratory Medicine, The First Affiliated Hospital of Nanjing Medical University, 210029 Nanjing, China; ^2^National Key Clinical Department of Laboratory Medicine, 210029 Nanjing, China

## Abstract

Current screening tests for the diagnosis of ovarian cancer (OC) face enduring challenges. However, microRNAs (miRNAs) are stable in the circulation and may be promising molecular biomarkers for OC prediction. Circulating miRNA expression profiles in OC were analyzed using sequencing data from the Gene Expression Omnibus database. Differentially expressed miRNAs were generated from GSE94533, of which some were selected as candidate miRNAs based on an electronic search of the literature and comprehensive evaluation. A meta-analysis was preformed to integrate an evaluation index for these miRNAs in diagnosing OC patients. An independent validation set (GSE106817) was also conducted to further confirm the roles of these miRNAs. We identified four *MIR200* members (*MIR200A*, *MIR200B*, *MIR200C*, and *MIR429*) and *MIR25* as being differentially expressed among malignant or benign ovarian tumor patients and healthy controls. In the meta-analysis, these five miRNAs yielded a pooled area under the receiver operating characteristic (ROC) curve (AUC) of 0.78 (sensitivity: 64%, specificity: 88%) in discriminating OC from healthy controls, while the four *MIR200* members demonstrated a summary AUC of 0.81 (sensitivity: 92%, specificity: 69%) in differing OC cases from patients with benign disease. In the validation set, differential expression and ROC curve analyses of these miRNAs were consistent except for *MIR25*. The circulating *MIR200* family has the potential to become reliable and noninvasive biomarkers for OC diagnosis. Studies with larger cohorts are warranted to validate the applicability of these miRNAs.

## 1. Introduction

Ovarian cancer (OC) is one of the most common and lethal gynecological malignancies and is the fifth leading cause of cancer death in females worldwide. Indeed, OC was reported to account for 2.6% and 5.0% of the total cancer incidence and deaths in women, respectively [1]. Most OC patients are diagnosed at late stages, and the 5-year survival rate is 46.5% based on the Surveillance, Epidemiology, and End Results Program, highlighting the need for an effective screening strategy. CA125 is the current serum biomarker for detecting and monitoring OC in clinical practice, but this is usually only elevated in advanced disease and has poorer sensitivity for early-stage cancer. Additionally, CA125 has limited specificity, and elevated levels do not always reflect a diagnosis of OC but can be indicative of endometriosis, liver cirrhosis, pelvic inflammatory disease, and some benign neoplasms [2, 3]. Thus, there is an urgent need to explore sensitive, noninvasive biomarkers for the detection of OC.

Accumulating evidence has shown that microRNAs (miRNAs) function as oncogenes or tumor suppressor genes. miRNAs are short (18–24 nucleotide) noncoding RNAs that regulate gene expression at the posttranscriptional level through base pairing with complementary sequences of the 3′-untranslated region of mRNA. Expression of miRNAs is aberrant in various types of cancers where they are readily detected in circulating body fluids such as serum or plasma [4]. Taylor and Gercel-Taylor previously isolated eight exosomal miRNAs (*MIR21*, *MIR141*, *MIR200A*, *MIR200B*, *MIR200C*, *MIR203*, *MIR205*, and *MIR214*) in the blood of OC patients that were upregulated in comparison with benign controls, which may reflect tumor profiles and aid diagnosis [5]. Following this investigation, a series of profiling studies were carried out to explore the diagnostic potential of extracellular miRNAs. Kan et al. identified a small marker panel combining *MIR200B* and *MIR200C* as a positive classifier of OC [6], while Langhe et al. showed that four miRNAs (*MIRLET7I*, *MIR122*, *MIR152*, and *MIR25*) were significantly downregulated in OC patients and could discriminate benign from malignant ovarian disease [7]. More recently, Elias et al. proposed a pattern of seven microRNAs (*MIR29A*, *MIR92A*, *MIR200C*, *MIR320C*, *MIR335*, *MIR450B*, and *MIR1307*) in the blood that appears to predict OC [8].

In this study, we profiled the global expression patterns of serum miRNAs in OC utilizing next-generation sequencing and clinical data from the Gene Expression Omnibus database (dataset ID: GSE94533). Considering the inconsistencies across different studies of the role of miRNAs in OC, we also performed systematic analysis combined with a validation study (GSE106817) to evaluate the diagnostic efficiency of circulating miRNAs with the aim of identifying a novel class of noninvasive biomarkers for the diagnosis of OC.

## 2. Materials and Methods

### 2.1. Next-Generation Sequencing Data and Differential Expression Analysis

miRNA profiling data were obtained from GSE94533 [8]. The corresponding platform was the Illumina NextSeq 500 system, and sequence tags were mapped to miRBase 20. Expression levels were quantified in transcripts per million. Using raw count data, the edgeR package of R was utilized to identify differentially expressed miRNAs (DEmiRNAs). A false positive rate < 0.05 and ∣log_2_FC∣ (fold change) > 1 were set as cutoff points to screen out DEmiRNAs.

### 2.2. Systematic Review and Meta-Analysis

We conducted a literature search for all studies that evaluated the diagnostic value of candidate circulating miRNAs for OC in the PubMed database up to January 2019. The following key terms were used in the search: “circulating” or “serum” or “plasma”, “DEmiRNAs screened above”, “Ovarian” and “cancer” or “carcinoma” or “neoplasm”. References cited in each retrieved article were also manually scanned to identify additional eligible studies.

Articles were recruited for this meta-analysis if they fitted the following criteria: (1) investigated the diagnostic value of circulating miRNAs in OC, (2) peripheral blood for miRNA analysis was collected before any treatment, (3) sufficient data to construct a 2 × 2 contingency table, and (4) published in English. The following data characteristics were collected for each included article: first author's name, publication year, country of publication, sample type, detecting method, sample size, and data for the two 2 × 2 contingency tables (sensitivity, specificity, area under the ROC curve (AUC), and corresponding 95% confidence interval (95% CI)).

### 2.3. Validation of Candidate miRNA Expression

The expression levels of candidate miRNAs were subsequently validated in an independent cohort (GSE106817) [9]. In this set, a total of 3938 serum samples were analyzed by miRNA microarray, including 320 OC, 2759 noncancer controls, and 859 other solid cancers. Comprehensive miRNA expression analysis was evaluated using the 3D-Gene® Human miRNA Oligo Chip (Toray Industries Inc., Tokyo, Japan). Each miRNA signal value was standardized using the ratio of the average signal value of the three internal control miRNAs (miR-149-3p, miR-2861, and miR-4463) to the preset value.

### 2.4. Functional Annotation and Enrichment Analysis for Candidate miRNAs

The potential targets of candidate DEmiRNAs were obtained using the miRTarBase database (http://mirtarbase.mbc.nctu.edu.tw/). We only included target genes that were validated by at least two of the following experimental methods: reporter assay, western blot, quantitative PCR, microarray, and next-generation sequencing experiments. Gene ontology (GO) enrichment and Kyoto Encyclopedia of Genes and Genomes (KEGG) pathway analysis were performed by the Database for Annotation, Visualization and Integrated Discovery (DAVID; https://david.ncifcrf.gov/) online tool. A Benjamini *P* value of <0.05 was used to indicate a statistically significant difference in the above pathway enrichment analysis.

### 2.5. Statistical Analysis

Data analysis was performed using Stata 13 (Stata Corporation, College Station, TX) and R (version 3.1.3) software. MIDAS modules for Stata were used to estimate the pooled specificity, sensitivity, and AUC of the summary receiver operating characteristic (SROC) [10]. Heterogeneity among studies was estimated with the *Q* test and *I* ^2^ statistics, and *I* ^2^ > 50% indicated the existence of significant heterogeneity. The presence of publication bias was detected using the Deeks funnel plot asymmetry test. Different distributions of relative serum miRNA expression levels between OC cases and controls in GSE94533 and GSE106817 were compared using the Mann–Whitney *U* test. ROC curve analysis was performed to calculate the AUCs to evaluate the associations of candidate miRNAs and OC. Tests of significance were two-tailed, and a *P* value < 0.05 was considered statistically significant.

## 3. Results

### 3.1. Differentially Expressed Serum miRNAs

**Figure 1:**
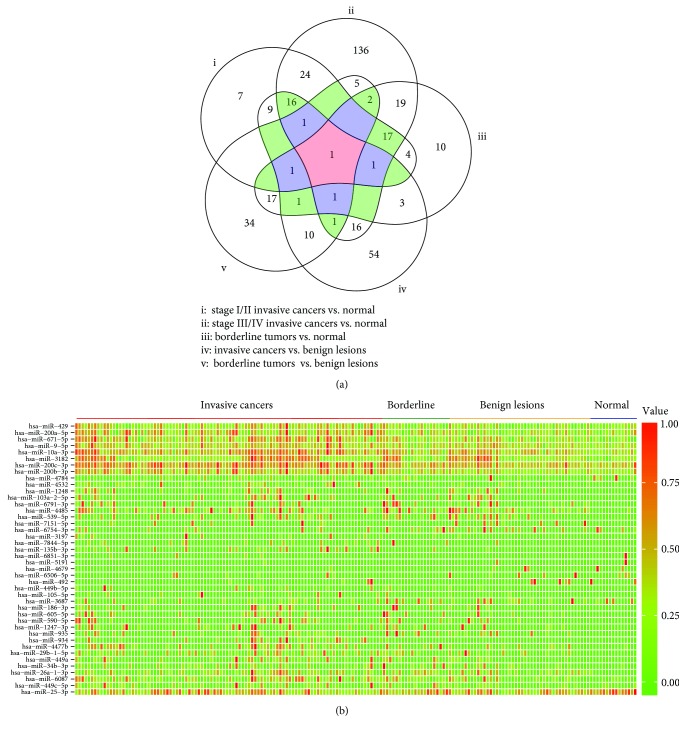
Identification of differentially expressed miRNAs in serum. (a) Five-set Venn diagram showing common differentially expressed miRNAs. (b) Heat map illustrating common miRNA profiles.

GSE94533 included 98 OC patients, 21 patients with borderline tumors, 45 patients with benign ovarian diseases, and 15 healthy women. Of the OC patients, 44.9% presented at stage III or IV disease, as defined by the International Federation of Gynecology and Obstetrics (FIGO). We used a five-set Venn diagram to demonstrate the common differentially expressed miRNAs (Figure 1(a)). A total of 81 DEmiRNAs were identified between stage I/II cancer and healthy controls, including 50 upregulated and 31 downregulated; 240 DEmiRNAs were identified between stage III/IV cancer and healthy controls, including 172 upregulated and 68 downregulated; 77 DEmiRNAs were identified between borderline tumors and healthy controls, including 52 upregulated and 25 downregulated; 115 DEmiRNAs were identified between invasive cancers and benign lesions, including 87 upregulated and 28 downregulated; and 74 DEmiRNAs were identified between borderline tumors and benign lesions, including 43 upregulated and 31 downregulated miRNAs. A total of 42 DEmiRNAs were common to three or more sets. Of these, 32 were upregulated and 10 were downregulated in invasive cancers or borderline tumors compared with benign lesions and healthy controls (Figure 1(b)).

### 3.2. Determination of Candidate miRNAs and Diagnostic Accuracy in OC

**Figure 2:**
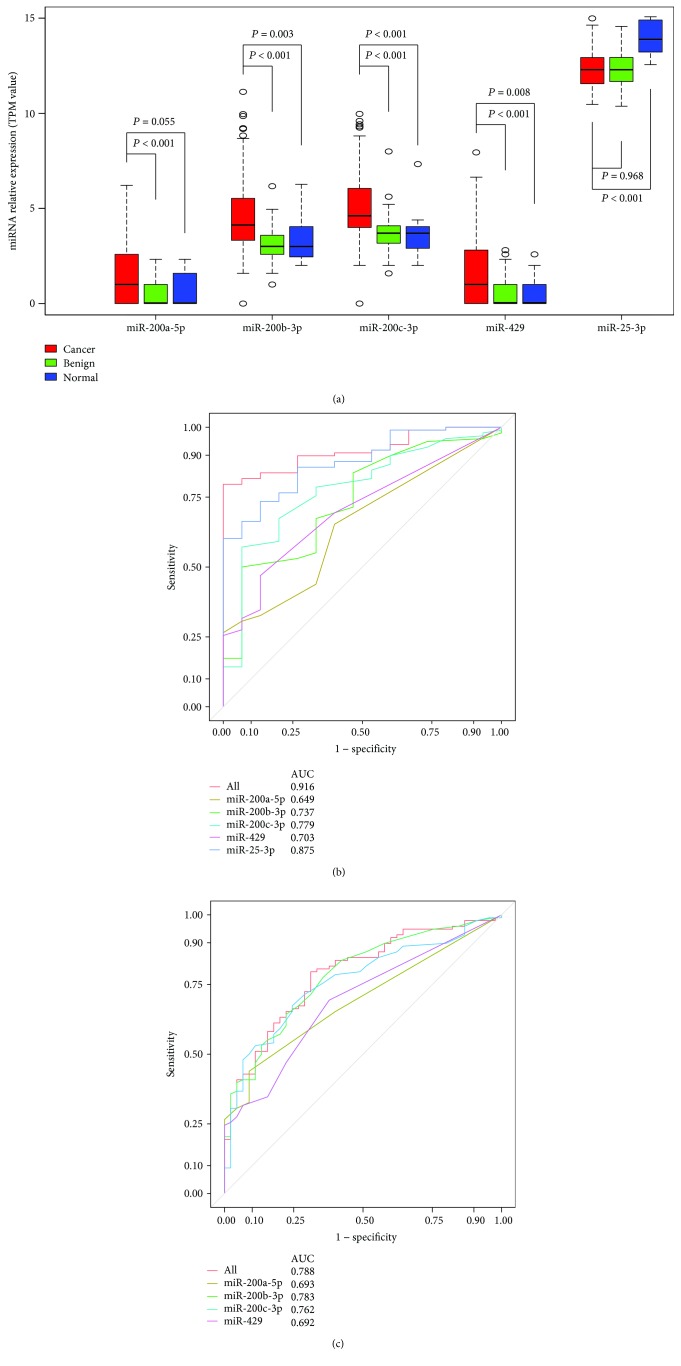
Quantification of candidate miRNAs in the serum of healthy women, patients with benign ovarian diseases, and those with ovarian cancer. (a) Box plot comparing miRNA concentrations in the serum of healthy women, patients with benign ovarian diseases, and those with ovarian cancer. (b) Receiver operating characteristic curve for candidate miRNAs showing its potential to discriminate ovarian cancer patients from healthy women. (c) Receiver operating characteristic curve showing the profiles of sensitivity and specificity of candidate miRNAs to distinguish ovarian cancer from benign ovarian diseases.

To further evaluate the clinical applicability of the 42 common DEmiRNAs for the diagnosis of OC, we screened all available studies based on the above search strategy and identified five miRNAs (*MIR200A*, *MIR200B*, *MIR200C*, *MIR429*, and *MIR25*) as being reported so far. We consequently focused our attention on their expression in GSE94533. As shown in Figure 2(a), miR-200b-3p, miR-200c-3p, and miR-429 serum expression was significantly higher in OC than in healthy controls, while the serum expression of miR-25-3p was extremely low in OC patients. Additionally, the expression of miR-200a-5p, miR-200b-3p, miR-200c-3p, and miR-429 was higher in OC patients compared with those with benign lesions.

To reflect the diagnostic potential of the selected miRNAs, ROC curve analysis was performed. We used information on the cutoff from each study to determine an optimal cutoff by maximizing the Youden index (sensitivity + specificity − 1). The relative expression of serum miR-200a-5p, miR-200b-3p, miR-200c-3p, miR-429, and miR-25-3p could distinguish OC patients from healthy controls with AUC values of 0.649, 0.737, 0.779, 0.703, and 0.875, respectively (Figure 2(b)). The significant differences in the serum levels of miRNAs between OC patients and patients with benign ovarian diseases were reflected by miR-200a-5p, miR-200b-3p, miR-200c-3p, and miR-429 AUC values of 0.693, 0.783, 0.762, and 0.692, respectively (Figure 2(c)). To improve the discrimination, the concentrations of these miRNAs were combined and analyzed by binary regression. One panel of these miRNAs could discriminate between OC patients and healthy controls with a sensitivity of 79.6%, a specificity of 100.0%, and an AUC value of 0.916 (Figure 2(b)); this panel could also differentiate OC from benign lesions with a sensitivity of 79.6% and a specificity and AUC value of 0.788 (Figure 2(c)).

### 3.3. Meta-Analysis of Serum miRNAs

To explore the diagnostic efficiencies of these miRNAs in OC, we performed a diagnostic meta-analysis. According to the search criteria, five articles were included in the meta-analysis [6, 8, 11–13] and their detailed information is listed in Table 1. In total, 562 OC patients, 159 healthy women, and 65 patients with benign ovarian diseases were included in the meta-analysis.

**Figure 3:**
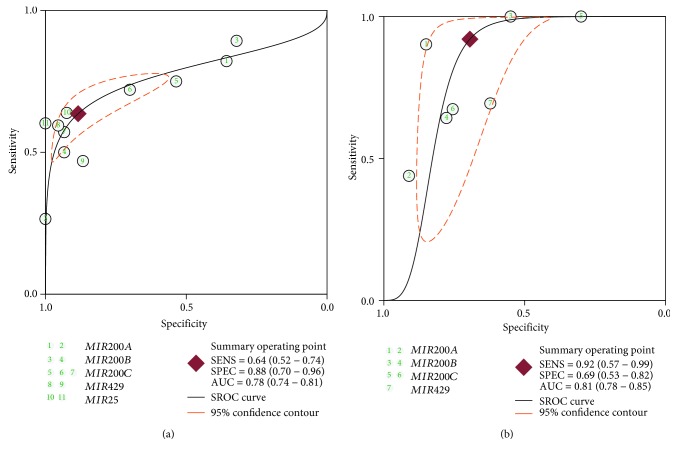
Summary receiver operating characteristic (SROC) curve for candidate miRNAs in the diagnosis of ovarian cancer for all studies. (a) SROC curve differentiating ovarian cancer from healthy controls. (b) SROC curve differentiating ovarian cancer from benign lesions.

To discriminate OC patients from healthy controls using the five miRNAs, we summarized sensitivity and specificity as 0.64 (95% confidence interval (CI): 0.52–0.74) and 0.88 (95% CI: 0.70–0.96), respectively; heterogeneity existed in both assessments (*P*_heterogeneity_ < 0.001; *I* ^2^ = 87.4 and 90.1%). The SROC plot showed the summary sensitivity and specificity and the 95% confidence and prediction regions, with an AUC of 0.78 (95% CI: 0.74–0.81, Figure 3(a)). To differentiate between OC and benign lesions (using *MIR200A*, *MIR200B*, *MIR200C*, and *MIR429*), the pooled sensitivity and specificity were 0.92 (95% CI: 0.57–0.99) and 0.69 (95% CI: 0.53–0.82), respectively. *I* ^2^ values for pooled sensitivity and specificity were 97.2% and 88.5%, respectively, indicating the existence of statistical heterogeneity between studies. Simultaneously, diagnostic accuracy was also assessed by SROC plotting, with an AUC value of 0.81 (95% CI 0.78–0.85, Figure 3(b)).

For our diagnostic meta-analysis, the funnel plot of publication bias showed no asymmetry for discriminating OC patients from healthy women and the Deeks test *P* value was 0.121. However, substantial funnel plot asymmetry suggestive of publication bias was revealed for differentiating OC from benign lesions (*P* = 0.002), possibly because only four articles containing seven studies were analyzed.

### 3.4. Candidate miRNA Analysis in the Validation Set

**Figure 4:**
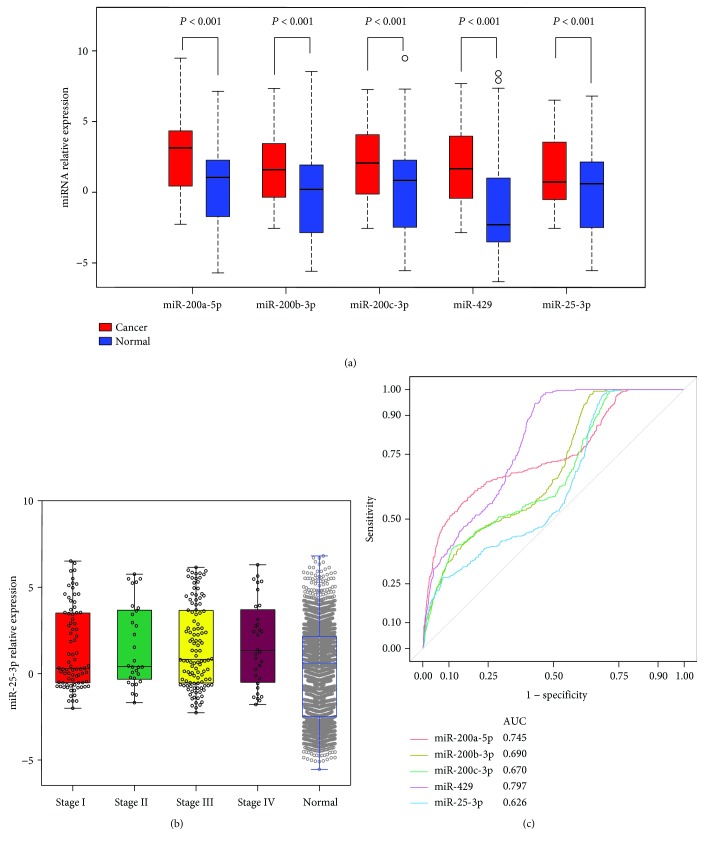
Quantification of candidate miRNAs in the validation set. (a) Box plot comparing miRNA concentrations in the serum of healthy women and patients with ovarian cancer. (b) The relative expression of miR-25-3p in the serum of healthy women and ovarian cancer patients stratified by tumor grade. (c) Receiver operating characteristic curve for candidate miRNAs showing its potential to discriminate ovarian cancer patients from healthy women.

To validate whether the five candidate miRNAs had potential as clinical biomarkers, we compared their expression in the independent set GSE106817. As shown in Figure 4(a), miR-200a-5p, miR-200b-3p, miR-200c-3p, and miR-429 showing significant alterations in expression were upregulated in OC with respect to the healthy control group (all *P* < 0.001). By contrast, miR-25-3p was significantly increased compared with healthy controls. When patients were stratified according to FIGO, miR-25-3p expression significantly decreased in stage I/II patients compared with controls (Figure 4(b)). These candidate miRNAs were also found to be aberrantly expressed in other solid tumors such as breast cancer, colorectal cancer, and gastric cancer (Figure S1). The diagnostic performance of the four miRNAs was confirmed in the validation set (AUC value: miR-200a-5p, 0.745; miR-200b-3p, 0.690; miR-200c-3p, 0.670; and miR-429, 0.797). miR-25-3p was calculated to have a sensitivity of 31.5%, a specificity of 75.5%, and an AUC of 0.626 (Figure 4(c)).

### 3.5. The Impact of Candidate miRNAs on Cellular Pathways and Biological Processes

**Figure 5:**
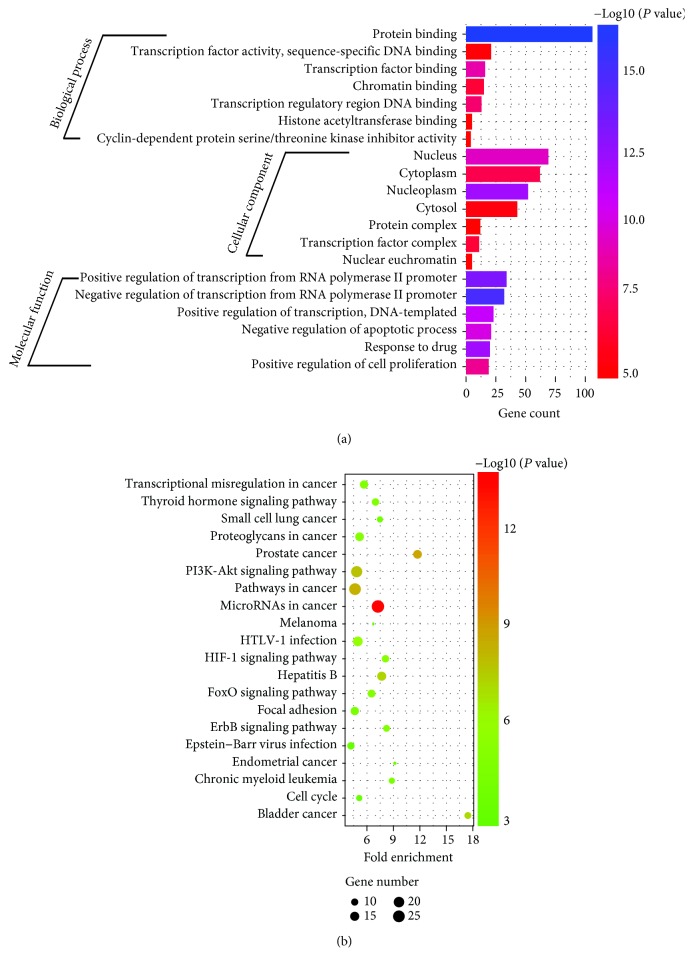
Functional annotation of the predicted targets of candidate miRNAs. (a) The top 20 GO terms derive from the “biological process,” “cellular component,” and “molecular function” categories by GO analysis. (b) The top 20 saturated pathways are generated from KEGG pathway analysis.

Experimentally validated targets from miRTarBase were extracted to elucidate the biological function of these candidate miRNAs, and 167 genes were found [14]. These genes were classified into three GO categories (biological process (BP), cellular component (CC), and molecular function (MF)) using the online analysis tool DAVID. BP genes exhibited significant enrichment in the negative/positive regulation of transcription from the RNA polymerase II promoter (GO:0000122/GO:0045944, *P* = 4.04 × 10^‐13^/2.90 × 10^‐11^). Among the CC and MF genes, the most clustered GO terms were nucleoplasm and protein binding, respectively (GO:0005654, *P* = 1.14 × 10^‐10^; GO:0005515, *P* = 2.17 × 10^‐15^). Regarding KEGG pathway enrichment analysis, the following three terms were identified as the most significant: miRNAs in cancer (hsa05206, *P* = 1.14 × 10^‐13^), prostate cancer (hsa05215, *P* = 4.68 × 10^‐8^), and pathways in cancer (hsa05200, 6.88 × 10^‐8^). The top 20 GO terms and enriched pathways are shown in Figure 5.

## 4. Discussion

There is emerging evidence that circulating miRNAs can be repeatedly and stably detected in the blood and serve as molecular markers in both physiological and pathological conditions for OC [15]. However, differences in measurement platforms, laboratory protocols, and small sample sizes can affect gene expression levels, so robust conclusions are rarely yielded across diverse studies. To compensate for these shortcomings, this study applied bioinformatics and meta-analysis to identify valuable circulating miRNAs in the diagnosis of OC.

Based on rigorous evaluations, we screened a total of 42 DEmiRNAs in GSE94533. Of these, *MIR200A*, *MIR200B*, *MIR200C*, *MIR429*, and *MIR25* have been previously reported. In the diagnostic meta-analysis, these five circulating miRNAs demonstrated a high diagnostic accuracy and yielded a combined AUC of 0.78 with 64% pooled sensitivity and 88% pooled specificity in discriminating OC cases from healthy controls. Excluding miR-25, the remaining four miRNAs showed a combined AUC of 0.81 with 92% pooled sensitivity and 69% pooled specificity in differentiating OC cases from patients with benign disease. Similarly, we observed the same diagnostic efficiency for these four miRNAs in our independent test.

The *MIR200* family contains *MIR200A*, *MIR200B*, *MIR200C*, and *MIR429*, which are generated from two distinct transcripts: *MIR200A*/*MIR200B*/*MIR429* is derived from chromosome 1 and *MIR200C* from chromosome 12 [6]. *MIR25* is located on chromosome 7 [16]. Members of the *MIR200* family are reported to be highly expressed in OC, as validated in our study, suggesting their importance in the diagnosis of OC. The *MIR200* family is thought to play an essential role in tumor metastasis by promoting epithelial-mesenchymal transition (EMT). *MIR200* family members help maintain E-cadherin expression in OC by downregulating ZEB1 and ZEB2, which are key transcription factors in EMT mediation that act as known repressors of E-cadherin transcription [17].

Decreased serum levels of *MIR25* were detected in GSE94533 in the present study, which is consistent with recent findings by Langhe et al. [7] and Meng et al. [11]. However, this phenomenon was only observed in early-stage OC samples in the GSE106817 set, which could reflect the different methods used for miRNA extraction and detection. Nevertheless, data on the effect of this miRNA in OC remain contradictory. miR-25 was previously reported to be significantly upregulated in OC compared with the healthy ovarian tissue [16, 18]. Additionally, *MIR25* was reported to interact with large numbers of protein-coding genes (*ITGA5*, *FBN1*, and *CDH1*) and noncoding genes (lncRNA *PTAF*) whose expression changes promote ovarian carcinogenesis [18, 19]. Interestingly, Benson et al. detected a significantly decreased circulating *MIR25* concentration (–1.82-fold) in OC patients who had undergone carboplatin chemotherapy compared to subjects before treatment, which indicated that its change was associated with clinical response [20].

To determine the potential target genes of these miRNAs, we conducted functional and signaling pathway analysis. The top 20 enriched GO terms and signaling pathways were shown to be involved in the development and prognosis of cancer, such as focal adhesion and the phosphoinositide 3-kinase/Akt signaling pathway. Many miRNAs have been found to influence this pathway which is considered to play an instrumental role in proliferation, migration, invasion, and chemotherapy resistance [21]. Focal adhesion is a common mechanism associated with tumor cell invasion and metastasis, including that of OC [22]. Therefore, taken together with previous findings, the varied functions of these miRNAs imply that they have the capacity to interact with several targets and effect mechanistic changes.

The expression of miRNAs in serum or plasma is fairly stable and can be detected using common laboratory methods. This may be because circulating miRNAs are protected by binding proteins or are chemically modified, such as by methylation, making them resistant to ribonuclease activity [23, 24]. Some researchers have proposed that circulating miRNAs are derived from the secretion or leakage of microvesicles, exosomes, or apoptotic bodies from healthy and tumor tissues [25]. This provides further support for OC-associated circulating miRNA expression profiles as indicators of biological function.

Our study has a number of strengths. The first is that we performed *in silico* analysis to investigate the diagnosis value and biochemical properties of circulating miRNAs in OC. Second, we included controls of patients with benign disease and healthy individuals. Third, the *MIR200* family with its high combined AUC and specificity was demonstrated to be a better molecular marker for OC prediction than *MIR25*, especially in the validation set. Fourth, we used a combination of bioinformatics and meta-analysis to identify OC biomarkers. However, we also recognize our study limitations, including the small sample size, selection bias, and heterogeneity across studies.

## 5. Conclusions

Our comprehensive analysis identified circulating *MIR200* family members as promising noninvasive screening tools for the early detection of OC. Further large-scale prospective studies are warranted to confirm the clinical relevance of these miRNAs.

## Figures and Tables

**Table 1:** Main characteristics of the studies included in the meta-analysis.

Author/publication year	Country	Sample	Method	Patients	Controls	miRNAs studied	AUC	Sensitivity	Specificity
Kan et al. [6]	Australia	Serum	Taqman	28 serous epithelial ovarian cancers	28 healthy women	*MIR200A* *MIR200B* *MIR200C*	0.675 0.722 0.727	0.821 0.893 0.750	0.357 0.321 0.536

Gao and Wu [13]	China	Serum	Taqman	74/19 epithelial/borderline ovarian cancers	50 healthy women	*MIR200C*	0.79	0.720	0.700

Meng et al. [11]	Germany	Serum	Taqman	180 epithelial ovarian cancers	66 healthy women	*MIR25* *MIR429*	0.834 0.704	0.924 0.594	0.639 0.955

Meng et al. [12]	Germany	Serum exosomes	Taqman	163 epithelial ovarian cancers	20 benign ovarian diseases	*MIR200A* *MIR200B* *MIR200C*	0.914 0.815 0.655	0.900 1.000 1.000	0.839 0.528 0.311

Elias et al. [8]	United States	Serum	Next-generation sequencing	98 epithelial ovarian cancers	15 healthy women	*MIR200A* *MIR200B* *MIR200C* *MIR429* *MIR25*	0.649 0.737 0.779 0.703 0.875	0.265 0.500 0.571 0.469 0.602	1.000 0.933 0.933 0.867 1.000
				98 epithelial ovarian cancers	45 benign ovarian diseases	*MIR200A* *MIR200B* *MIR200C* *MIR429*	0.693 0.783 0.762 0.692	0.439 0.643 0.673 0.694	0.911 0.778 0.756 0.622

## Data Availability

The raw data of next-generation sequencing in this study are available in the NCBI database (https://www.ncbi.nlm.nih.gov/geoprofiles/) under accession number GSE94533. The microarray data that support this study are available through the NCBI database under accession GSE106817. The data used to support the findings of this study are included within the article.

## Supplementary Materials

Supplementary Materials Figure S1: The relative expression levels of 5 identified miRNAs in serum samples from patients with different types of tumors and healthy women: (a) miR-200a-5p, (b) miR-200b-3p, (c) miR-200c-3p, (d) miR-429, and (e) miR-25-3p.
