# Supplementary material for: In Silico Screening of Circulating MicroRNAs as Potential Biomarkers for the Diagnosis of Ovarian Cancer
Source: Dis Markers. 2019 Aug 4;2019:7541857. doi: 10.1155/2019/7541857 (PMC6701281; doi:10.1155/2019/7541857)

Supplementary Materials

Figure S1: The relative expression levels of 5 identified miRNAs in serum samples from patients with different types of tumors and healthy women. (a) miR-200a-5p, (b) miR-200b-3p, (c) miR-200c-3p, (d) miR-429, (e) miR-25-3p.

Figure S1

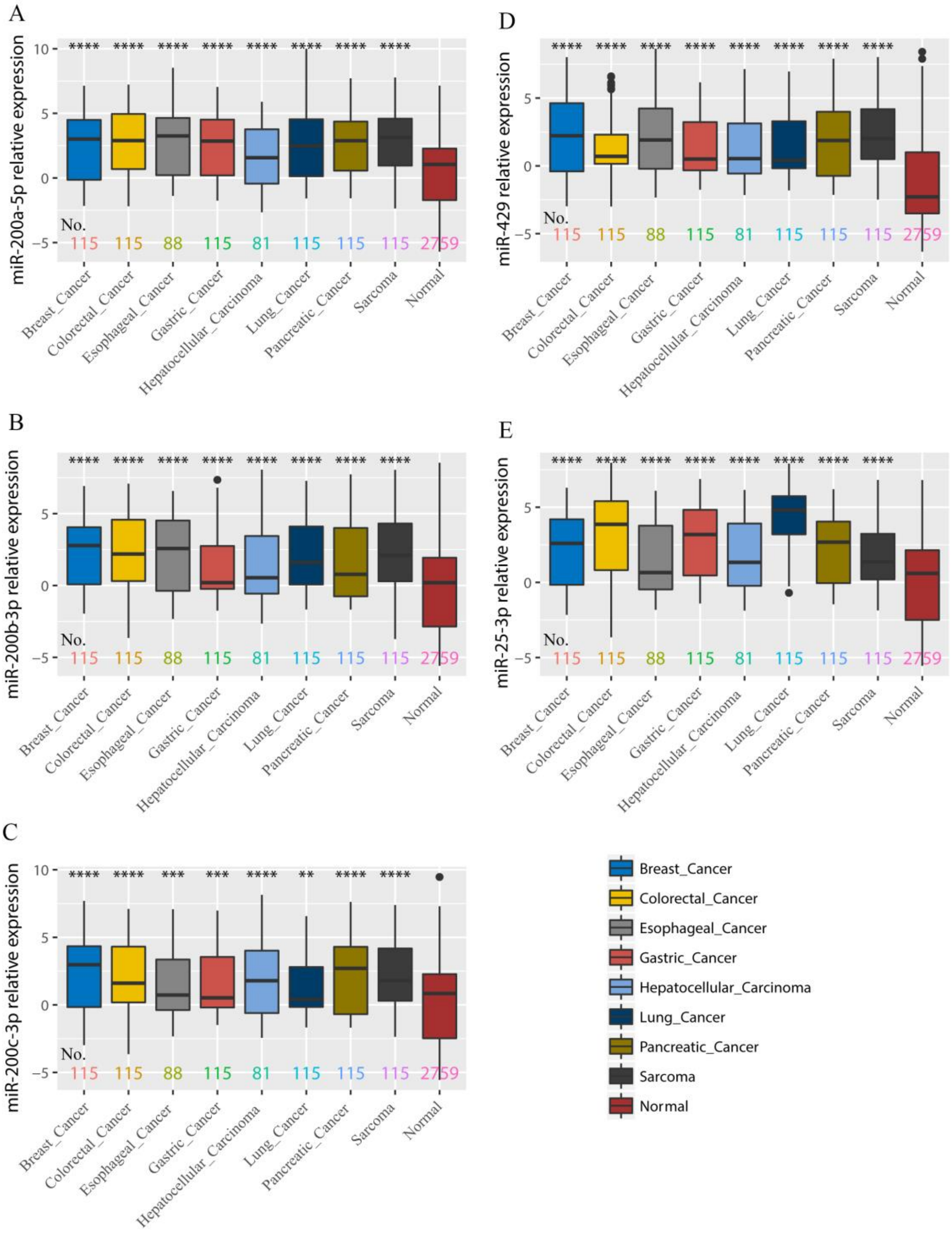

Supplement: Supplementary Materials — Figure S1: The relative expression levels of 5 identified miRNAs in serum samples from patients with different types of tumors and healthy women: (a) miR-200a-5p, (b) miR-200b-3p, (c) miR-200c-3p, (d) miR-429, and (e) miR-25-3p. [file 7541857.f1.pdf]
